# Supplementary figures and images for: Circulating Blood-Brain Barrier Proteins for Differentiating Ischaemic Stroke Patients from Stroke Mimics
Source: Biomolecules. 2024 Oct 22;14(11):1344. doi: 10.3390/biom14111344 (PMC11592266; doi:10.3390/biom14111344)

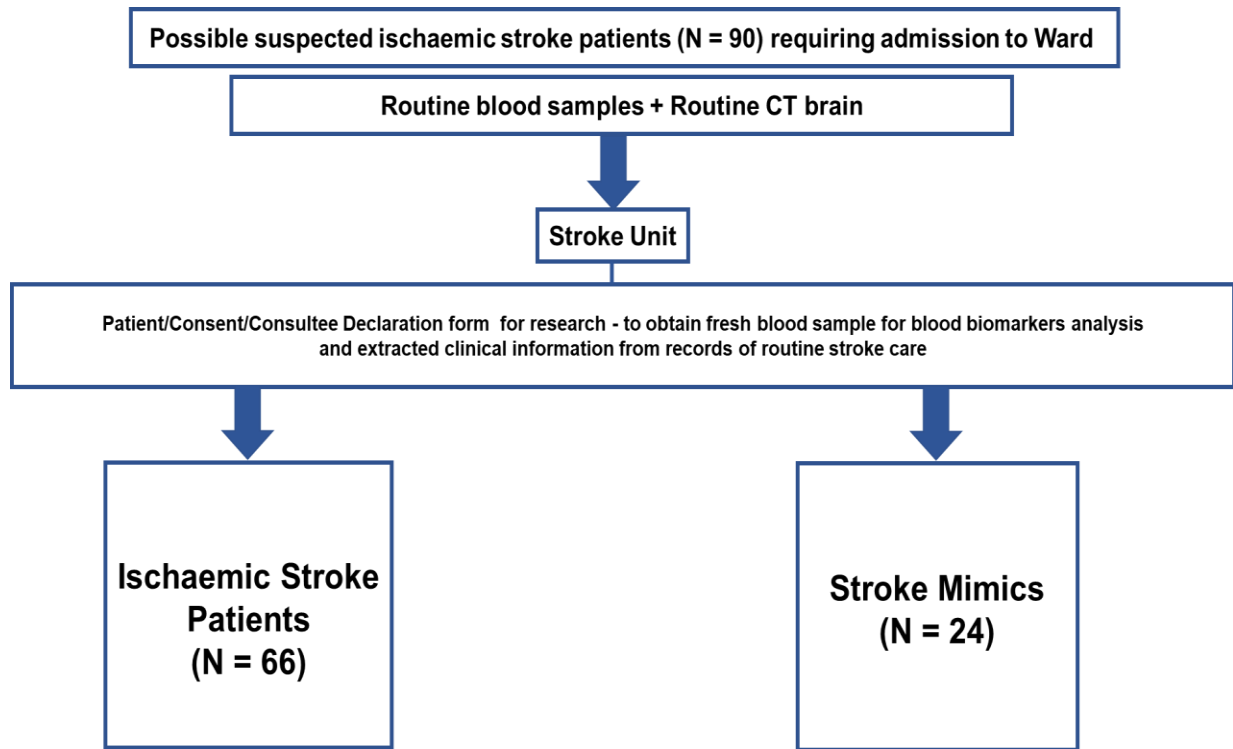

**Figure S1: Flow diagram.** Design of the study and patients' routine clinical pathways.

Supplement: Supplementary file 1 [file biomolecules-14-01344-s001.zip › Figure S1.pdf]
